# Binding of CCCTC-Binding Factor (CTCF) to the Minute Virus of Mice Genome Is Important for Proper Processing of Viral P4-Generated Pre-mRNAs

**DOI:** 10.3390/v12121368

**Published:** 2020-11-30

**Authors:** Maria Boftsi, Kinjal Majumder, Lisa R. Burger, David J. Pintel

**Affiliations:** 1 Pathobiology Area Graduate Program, Christopher S. Bond Life Sciences Center, School of Medicine, University of Missouri-Columbia, Columbia, MO 65211, USA; boftsim@umsystem.edu; 2 Christopher S. Bond Life Sciences Center, Department of Molecular Microbiology and Immunology, School of Medicine, University of Missouri-Columbia, Columbia, MO 65211, USA; kmajumder@wisc.edu (K.M.); burgerlr@missouri.edu (L.R.B.)

**Keywords:** parvovirus, minute virus of mice, RNA processing, gene expression

## Abstract

Specific chromatin immunoprecipitation of salt-fractionated infected cell extracts has demonstrated that the CCCTC-binding factor (CTCF), a highly conserved, 11-zinc-finger DNA-binding protein with known roles in cellular and viral genome organization and gene expression, specifically binds the genome of Minute Virus of Mice (MVM). Mutations that diminish binding of CTCF to MVM affect processing of the P4-generated pre-mRNAs. These RNAs are spliced less efficiently to generate the R1 mRNA, and definition of the NS2-specific exon upstream of the small intron is reduced, leading to relatively less R2 and the generation of a novel exon-skipped product. These results suggest a model in which CTCF is required for proper engagement of the spliceosome at the MVM small intron and for the first steps of processing of the P4-generated pre-mRNA.

## 1. Introduction

Parvoviruses are small (20 nm) non-enveloped icosahedral viruses that infect and cause disease in many vertebrate hosts. They are unique among all known animal viruses in that they contain ~5 kb single-stranded linear DNA genomes, with inverted terminal repeats at both ends, which form hairpin structures and serve as origins of replication [1].

The viral genome is organized into two overlapping transcription units, producing three major transcript classes, R1, R2, and R3 [2]. Transcripts R1 (4.8 kb) and R2 (3.3 kb), generated from a promoter (P4) near the left-hand end of the genome, encode the two non-structural proteins NS1 (83 kDa) and NS2 (24 kDa), respectively [3]. Transcripts R3 (3.0 kb), generated from P38 promoter, encode the two viral capsid proteins, VP1 (83 kDa) and VP2 (64 kDa), utilizing the open reading frame (ORF) in the right half of the genome [4,5]. A small, overlapping intron, common to both P4- and P38-generated transcripts, utilizes two donors, D1 (nt 2280) and D2 (nt 2317) and two acceptors, A1 (nt 2377) and A2 (nt 2399), which are alternatively used to produce nine different spliced mRNA species [2,6,7,8]. A large upstream intron, present in the pre-mRNAs generated from the P4 promoter, is either retained (R1 transcript class) or excised (R2 transcript class) in mature mRNAs [9].

Upon initiation of parvovirus replication, MVM forms distinct foci in the nucleus termed autonomous parvovirus-associated replication (APAR) bodies where active transcription of viral genes and viral replication takes place [10]. The viral replicator protein NS1 co-localizes with the replicating viral genome in APAR bodies, where DDR sensor and response proteins, host replication factors, and cell cycle regulators also reside [10,11,12].

Using a novel adaptation of high-throughput chromosome conformation capture assay, V3C (Viral Chromosome Conformation), sites on the cellular genome where MVM localizes for replication have been mapped. These cellular regions preferentially accrue DNA damage in uninfected as well as MVM infected cells, and are also constituent parts of chromosomal substructures called Topologically Associating Domains (TADs) [13,14]. These are large, megabase-sized genomic regions, which are defined by preferential interactions within them and thus are relatively insulated from neighboring regions [15,16]. The boundaries of TADs are enriched for binding sites of CCCTC-binding factor (CTCF), a highly conserved, 11-zinc-finger DNA-binding protein, which along with cohesin, play a key role in the formation and maintenance of topological domains [15,17,18]. In addition to its role in genome organization, CTCF regulates key aspects of gene expression, including transcriptional activation/repression, and enhancer/promoter insulation, by facilitating long-range chromatin interactions via looping [19,20]. Apart from its DNA-binding activity, it was reported that CTCF can bind RNA and that CTCF-RNA interactions can participate in CTCF-mediated chromatin loop formation and subsequent regulation of gene expression [21,22]. Emerging evidence suggests that CTCF also regulates gene expression at the level of mRNA splicing. More specifically, CTCF has been shown to promote inclusion of weak upstream exons in the mRNA of CD45 gene by mediating local RNA polymerase II pausing [23]. Moreover, a more recent study showed that CTCF-mediated intragenic chromatin looping facilitates inclusion of exons in spliced mRNA by bringing exons in physical proximity, providing a functional link between chromatin organization and regulation of splicing [24].

It has become clear that a number of viruses, including Kaposi’s sarcoma-associated herpesvirus (KHSV), Epstein-Barr virus (EBV), and human cytomegalovirus (HCMV), utilize CTCF to control viral gene expression [25,26,27]. It was demonstrated that CTCF associates with several regions within the KHSV genome, and that the CTCF-cohesin protein complex regulates the cell cycle control of viral gene expression during latency [25]. In a later study, it was also shown that CTCF and cohesin play important roles in regulating KHSV reactivation from latency by modulating viral gene transcription [28]. CTCF binding on EBV genome was shown to negatively affect transcription [26] and in the case of HCMV, binding of CTCF to the first intron of the Major Immediate Early (MIE) gene repressed MIE gene expression [27]. In addition, CTCF recruitment to the small DNA genome of human papillomavirus (HPV) was shown to regulate viral gene expression and transcript processing [29].

In this report, we show that CTCF can play an important role in parvovirus gene expression. Mutations that diminish binding of CTCF to the MVM genome affect processing of P4-generated pre-mRNAs; R1 is spliced less efficiently, and definition of the NS2-specific exon upstream of the small intron is reduced, leading to relatively less R2 and the generation of a novel exon-skipped product. These results implicate a requirement for CTCF in engagement of the spliceosome at the MVM small intron and the first steps of processing of the P4-generated pre-mRNA.

## 2. Materials and Methods

### 2.1. Cell Lines and Viruses

Murine A9 and human NB324K cells were maintained in Dulbecco’s modified Eagle’s medium (DMEM) supplemented with 5% fetal bovine serum (FBS) and incubated at 37 °C with 5% CO_2_. Wild-type MVMp for infections was produced in A9 cells as previously described [11].

### 2.2. Transfections and Viral Infections

For transfections, cells were grown on 60-mm tissue culture dishes until they reached ~80% confluency. Cells were transfected with plasmids using LipoD293 transfection reagent (SignaGen Laboratories, Baltimore, MD, USA) according to the manufacturer’s instructions. For RNA isolation, cells were co-transfected with wild-type or mutant plasmids (2 μg) and 3xFLAG-eGFP (p3xFeGFP) expression vector (0.5 μg), and harvested at 48 h post transfection (hpt). For Chromatin Immunoprecipitation (ChIP) assays, cells were transfected with 2 μg of wilt-type or mutant plasmids and harvested at 20 hpt. Viral infections were carried out at a Multiplicity of Infection (MOI) of 10 unless otherwise stated and infected cells were harvested at the indicated timepoints.

### 2.3. Cell Synchronization

For infection experiments, A9 cells were parasynchronized in G0 phase by isoleucine deprivation for 36–42 h prior to infection as previously described [11]. Following synchronization, cells were released into complete media containing 5% FBS and infected with MVMp.

### 2.4. Plasmids

The infectious plasmid clone of MVM (pWT), which expresses the full length viral genome, was previously described [30]. gBlocks Gene Fragments of MVM, gbNSc and gbVPc, containing the mutated CTCF binding sites at the NS and VP region, respectively, were synthesized by Integrated DNA Technologies (IDT, Coralville, IA, USA). pNSc plasmid was constructed by replacing the XcmI-BsrGI fragment of pWT (nt 644–1253) with the gbNSc gene block, so that MVMp with a mutated CTCF binding site at the NS region was expressed. Similarly, the pVPc plasmid was constructed by replacing the PmlI-XbaI fragment of pWT (3636–4347) with the gbVPc gene block, so that MVMp with a mutated CTCF binding site at the VP region was expressed. In order to make the double CTCF-binding site mutant plasmid, pDc, in which both CTCF binding sites were mutated, the PmlI-XbaI fragment in pNSc was replaced with the gene block gbVPc. To generate the marker rescue of the pNSc plasmid, pNScMR, the XcmI-BsrGI fragment in pNSc was replaced with the XcmI-BsrGI fragment from pWt. The marker rescue of the pDc plasmid, pDcMR, was constructed by replacing both the XcmI-BsrGI and PmlI-XbaI fragments in pDc with the corresponding fragments from pWT. The p4Tppt plasmid, with improved polypyrimidine tract at the large intron 3′ splice site, was previously described [31]. To generate the double CTCF-binding site mutant construct with improved polypyrimidine tract (pDc4Tppt), the BsrGI-XhoI fragment (nt 1248–2075) from p4Tppt was cloned into the pDc plasmid between the BsrGI and XhoI sites. The pD3 plasmid was constructed by replacing both the XcmI-BsrGI and PmlI-XbaI fragments in pWT with the gene blocks gbNS3 and gbVP3, respectively.

### 2.5. Extraction of MVMp Nucleoprotein Complexes

MVMp nucleoprotein complexes were isolated from infected cells as previously described with modifications [32]. At the indicated timepoints, cells were washed with phosphate-buffered saline (PBS), harvested into HBE buffer (10 mM HEPES, 5 mM KCl, 1 mM EDTA), and collected by centrifugation at 1000× *g* for 3 min. Cell pellets were resuspended in 500 μL HBE buffer and lysed on ice for 10 min by addition of 1% NP-40 (to a final concentration of 0.1%). To pellet the nuclei, the lysate was centrifuged for 5 min at 1000× *g*. The supernatant (cytoplasmic extract) was transferred to a clean tube and the nuclei was resuspended in 500 μL buffer HBE. Sodium chloride (NaCl) was added to the suspension to a final concentration of 100, 200, or 400 mM and incubated on ice for 2 h. The remaining chromatin (chromatin pellet) was pelleted at 10,000× *g* for 10 min while the supernatant contained the MVMp nucleoprotein complexes (salt-wash extract).

### 2.6. Total RNA Isolation

Total RNA was extracted from transfected or infected cells as previously described with minor modifications [33]. Briefly, for total RNA isolation, cells were lysed in TRIzol reagent (Invitrogen, Carlsbad CA, USA) and RNA was prepared according to the manufacturer’s protocol.

### 2.7. RNase Protection (RPA) Assay

Total RNA was extracted from transfected or infected cells using TRIzol reagent (Invitrogen) according to the manufacturer’s protocol and RNase protection assays were performed on 25 μg RNA as previously described [34]. The probes used for the RPAs were α-^32^P-UTP-labeled Sp6-generated antisense RNAs. The MVM HaeIII probe, extended from before the acceptor site of the large intron (nt 1852) to within the small intron (nt 2378), was used to analyze all MVM pre-mRNAs generated during wild-type MVMp infection. The HaeIII fragment (nt 1852–2378), cloned into a pGEM-3Z cloning vector between the XbaI and SphI restriction sites, was used as a template for the preparation of the HaeIII probe. Appropriate homologous probe (HaeIII 4Tppt) was used to analyze the RNA species generated from the Dc4Tppt mutant. The MVM P4 probe (spanning nt 201 to 652) was produced to analyze the P4-generated RNA products. The MVM 201–652 fragment was cloned into the pGEM-3Z vector between the BamHI and HindIII restriction sites and it was used as a template for the synthesis of the P4 probe.

To make the 3xFeGFP antisense RNA probe, the 3xFeGFP fragment with a SP6 promoter sequence at the 3′ end was amplified from the 3xFeGFP expression vector (p3xFeGFP) by PCR with primers 5′ ATC ATG CGG CCG CCG TCA GAA TTA ACC ATG GAC TAC AAA GAC 3′ and 5′ CTA TAT TTA GGT GAC ACT ATA GTT AAT TTT ATT AGG ACA AGG CTG GTG 3′.

### 2.8. Northern Blotting

For Northern blot analysis, 10 μg of total RNA, prepared as described above, was resolved on a formaldehyde—1.4% agarose gel at 35 mA for 24 h. After staining with ethidium bromide for 30 min, the gel was washed in DEPC-treated water for 4 h and transferred to a nitrocellulose membrane overnight. Blots were baked for 2 h at 80 °C and hybridized with randomly primed radiolabeled MVM probes. A HaeIII probe (nt 1852–2378) was used to detect all full-length viral mRNAs and a whole genome probe (Bam) was used to specifically detect the exon-skipped product generated from the double CTCF-binding site mutant construct.

### 2.9. Chromatin Immunoprecipitation (ChIP) Assay in Whole Cell Lysates

ChIP assays were conducted on parasynchronized murine A9 cells infected with MVMp at an MOI of 10 or human NB324K cells transfected with the wild-type or the CTCF-binding site mutant constructs as described previously [13]. Briefly, cells were cross-linked by addition of 1% formaldehyde directly to the culture media and incubated with shaking at room temperature for 10 min. The reaction was quenched with 0.125 M glycine for 5 min and cells were collected and lysed for 20 min on ice in ChIP lysis buffer (1% SDS, 10 mM EDTA, 50 mM Tris-HCl pH 8.0, protease inhibitors). Cell lysates were sonicated with a Diagenode Bioruptor for 75 cycles (30 s on and 30 s off) and debris was pelleted by centrifugation (8000× *g*, 15 min, 4 °C). The supernatant was then added to the indicated antibody-bound Protein A Dynabeads (Invitrogen) and samples were incubated overnight with rotation at 4 °C in ChIP dilution buffer (0.01% SDS, 1.1% Triton X-100, 1.2 mM EDTA, 16.7 mM Tris-HCl pH 8.0, 167 mM NaCl). The next day, the following washes were performed (3 min each at 4 °C with rotation): once in low salt wash (0.01% SDS, 1% Triton X-100, 2 mM EDTA, 20 mM Tris-HCl pH 8.0, 150 mM NaCl), once in high salt wash (0.01% SDS, 1% Triton X-100, 2 mM EDTA, 20 mM Tris-HCl pH 8.0, 500 mM NaCl), once in lithium chloride (LiCl) wash (0.25 M LiCl, 1% NP40, 1% DOC, 1 mM EDTA, 10 mM Tris-HCl pH 8.0) and twice in TE buffer, followed by elution in SDS elution buffer (1% SDS, 0.1 M Sodium bicarbonate). The DNA-antibody complexes and input DNA were reverse cross-linked overnight at 65 °C in the presence of NaCl and proteinase K. The DNA was purified using a PCR purification kit (Qiagen, Baltimore, Maryland, USA) and analyzed by quantitative PCR (qPCR) with iTaq universal SYBR green master mix (Bio-Rad) and primers 5′ CGC CTT CGG ACG TCA CAC GTC 3′ (MVM nt 60–80) and 5′ CCA GCC ATG GTT AGT TGG TTA C 3′ (MVM nt 268–247). Data are presented as percent input, calculated as described previously, ref. [35] or relative to IgG.

### 2.10. Chromatin Immunoprecipitation (ChIP) Assay on Viral Nucleoprotein Complexes

Following the extraction of MVMp nucleoprotein complexes as described above, the salt-wash extract was cross-linked with 0.1% formaldehyde for 5 min at room temperature and the reaction was quenched with 0.125 M glycine. The sample was then loaded onto an Amicon Ultra-0.5 Centrifugal Filter Device placed in filtrate collection tube and centrifuged for 30 s at 10,000× *g* to remove salt. PBS was added to the remaining sample and centrifuged for 30 s at 10,000× *g* to exchange buffer and concentrate. The purified sample was recovered from the Amicon filter by reverse spin (1000× *g*, 2 min). The viral genome-protein complexes were incubated with the indicated antibodies bound to Dynabeads Protein A (Invitrogen) and the ChIP assay performed as described above.

### 2.11. Immunoblot Analysis

Infected cells were harvested at the indicated timepoints, lysed in 1× dye (25 mM Tris pH 7.5, 2% SDS, 2 mM EDTA, 6% glycerol, 20 mM DTT, bromophenol blue) and sheared using a 25 G × 5/8-inch, 1-mL needle-syringe (BD Biosciences San Jose, CA, USA). The whole-cell lysates were boiled for 10 min at a 100 °C-heat block and equal volumes of samples were loaded per well for Western blot analysis. For Western blot analysis of the salt-wash extracts, 1× dye was added directly to the samples and processed as described above. Chromatin pellet, prepared during the salt-wash extraction procedure, was resuspended in 1× dye, sheared, and processed as described above.

### 2.12. Southern Blot Analysis

Infected cells were harvested at the indicated timepoints, pelleted and resuspended in Southern lysis buffer (2% SDS, 150 mM NaCl, 10 mM Tris pH 8.0, 1 mM EDTA). Cells were proteinase K treated for 2 h at 37 °C, and sheared using 25 G × 5/8-inch, 1-mL needle-syringe (BD Biosciences). Total DNA content in the samples was quantified using Nanodrop, equal amount of DNA loaded per well and electrophoresed on a 1% agarose gel for 16 h at 35 V. Samples were transferred to a nitrocellulose membrane and hybridized with randomly primed radiolabeled MVM probe (Bam) or genomic DNA probe (SINE). For Southern blot analysis of the chromatin pellet and salt-wash extracts, samples were resuspended in Southern lysis buffer and processed as described above.

### 2.13. Reverse Transcription-Polymerase Chain Reaction (RT-PCR) and TA Cloning

Total RNA was extracted using TRIzol reagent (Invitrogen) from cells transfected with the wild-type (pWT) and the double CTCF mutant (pDc) vectors, respectively, and subjected to DNase I (Thermo Fisher Scientific, Waltham MA, USA) treatment for 1 h at 37 °C to remove genomic DNA contamination. First-strand cDNA synthesis was performed on 1 μg DNase I-treated RNA using SMART^®^MMLV Reverse Transcriptase (Clontech, Mountain View, CA, USA) according to the manufacturer’s instructions with primer 5′ GTT TTT TTT TAG CTC TGG CTT GG 3′ (MVM 2758–2736). The cDNA product was used for downstream PCR amplification using Platinum™ Taq DNA Polymerase High Fidelity (Invitrogen) with primers 5′ GTA TTG ATC ATA GGC CTC GTC G 3′ (MVM 2514–2493) and 5′ GTA ACC AGG AAG TGT TCT CAT TTG 3′ (MVM 322–345). The PCR products were analyzed by agarose gel electrophoresis and individual bands were extracted from the gel for downstream analysis using the QIAquick Gel Extraction kit (Qiagen). The small product, generated from the double CTCF-binding site mutant construct, was cloned into a PCR^®^2.1 vector using the TA Cloning^®^ Kit (Invitrogen) according to the manufacturer’s protocol. The construct was transformed into competent *Escherichia coli* DH5α cells and a number of individual clones were analyzed by Sanger Sequencing. The large product, generated from both the wild-type and the mutant construct, was submitted directly for sequencing analysis.

### 2.14. Immunofluorescence Assay

Immunofluorescence assays were performed in human NB324K cells infected with MVMp at an MOI of 10. At 24 h post infection (hpi), cells were harvested and processed as previously described [13]. Samples were incubated with the indicated antibodies for 1 h followed by the Alexa Fluor^®^ conjugated secondary antibodies 488 and 568 for 1 h. Samples were mounted on slides with ProLong Diamond Artifade Mountant with DAPI (Invitrogen) and images were acquired using a Leica TCP SP8 confocal microscope and a 10 × 1.4 NA objective lens.

## 3. Results

### 3.1. CTCF Specifically Binds the Viral Genome and Localizes to MVM Replication Compartments

**Figure 1:**
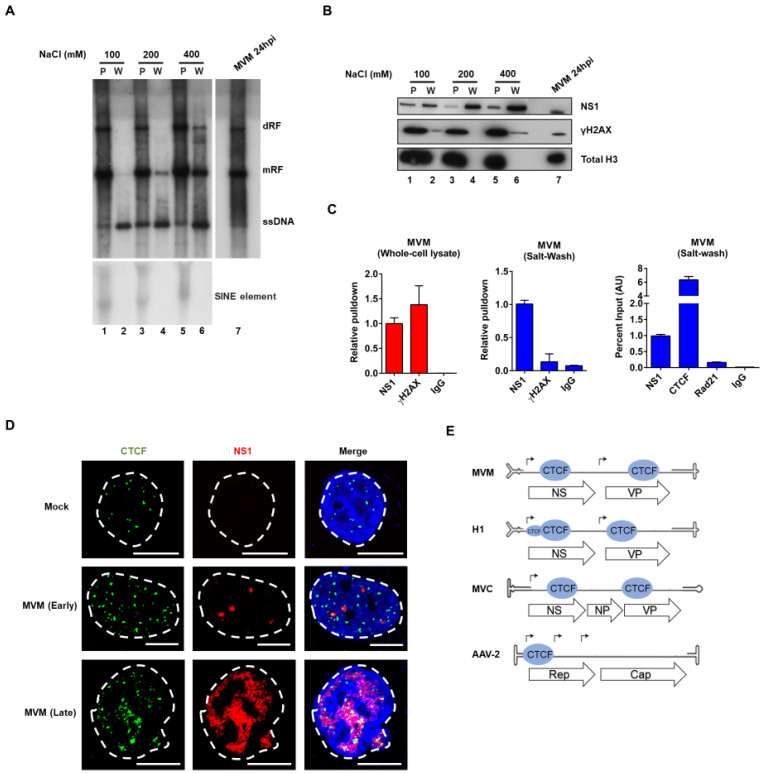
CTCF is associated with the viral genome and localizes to MVM replication compartments. (**A**) Murine A9 cells were parasynchronized by isoleucine deprivation and infected with MVMp at an MOI of 10 at the time of release into complete medium. At 24 hpi, viral nucleoprotein complexes were extracted from infected cells with various NaCl concentrations, and both the chromatin pellet (P) and the salt-wash extracts (W) were subsequently analyzed by Southern blotting as described in Materials and Methods. DNA extracted directly from infected cells served as a positive control (lane 7). The blot was hybridized with a radiolabeled MVM whole genome probe (top panel) and replicative intermediates of single-stranded DNA, ssDNA; monomer, mRF; and dimer, dRF; are indicated to the right. The blot was also hybridized with a genomic DNA probe against the SINE element (bottom panel). (**B**) Salt wash extracts and chromatin pellet described in (**A**) were assayed by Western blotting using antibodies directed against the indicated proteins. Whole-cell lysates of MVM-infected parasynchronized murine A9 cells were also analyzed by Western blotting with the indicated antibodies and served as a positive control (lane 7). (**C**) Murine A9 cells were parasynchronized by isoleucine deprivation and infected with MVMp at an MOI of 10 at the time of release into complete medium. At 16 hpi, cells were processed, as described in Materials and Methods for whole-cell lysate ChIP (left panel) or ChIP on salt wash extracts (middle and right panel) with the indicated antibodies. Samples were analyzed by qPCR as described in Materials and Methods. Data are presented as mean ± standard error of the means (SEM) of two individual experiments. Background binding levels were determined using mouse IgG pulldowns. (**D**) Representative confocal images of Mock versus MVM infected, non-synchronized human NB324K cells at 24 hpi, probing MVM-NS1 (red) and the host cellular factor CTCF (green). CTCF co-localized with NS1 in both early and late stage APAR bodies designated as previously described [12] (middle and bottom panel respectively). Blue corresponds to DAPI staining. Nuclear border is indicated by dashed white line. (**E**) Schematic representation of the protoparvoviruses MVM, MVC, H1, and the dependovirus AAV2 genome showing the positions of transcriptional promoters (solid black arrows), the major open reading frames that encode the viral non-structural and capsid proteins (arrowed boxes), and the relative positions of CTCF binding sites (blue oval shapes).

We previously showed that MVM replicates in close association with sites on the cellular genome, taking advantage of the fact that these sites are replete with factors involved in gene expression and DNA damage signaling [13]. Therefore, in order to identify and characterize factors that specifically bound the MVM genome during replication, we developed a nuclear salt-wash extraction protocol, which could effectively separate the replicating viral genome from cellular DNA prior to cross-linking. This protocol, based on previous strategies designed to purify soluble nuclear protein complexes of MVM [32], was here further optimized across both time and salt gradients. Two-hour incubations proved best, and as can be seen in Figure 1A, MVM replicative forms were efficiently extracted beginning at approximately 200 mM NaCl. Both histone H3 and γ-H2AX, typically associated with cellular DNA [36], were used to monitor the purity of fractionation following extraction. As can be seen in Figure 1B, γ-H2AX appeared in the 400 mM salt-wash (the γ-H2AX band in the 100 mM salt-wash was not reproducible, and likely was an overflow from the adjacent lane), and so subsequent experiments were performed using extraction conditions of 200 mM NaCl for 2 h.

In silico inspection of the MVM genome suggested a potential interaction with the multifunctional cellular DNA-binding zinc finger protein CTCF, and thus this potential interaction was investigated by ChIP assays of salt-wash extracts of MVM infected cells [37]. First, the purity of the salt-wash extraction as assayed by ChIP was confirmed. While γ-H2Ax was found to associate strongly with MVM by ChIP following cross-linking within total cell extracts (Figure 1C, left panel), γ-H2Ax did not bind the MVM genome significantly over background when cross-linking and ChIP assays were performed in optimized salt-wash extracts (Figure 1C, center panel). These results were consistent with the results of the Western blot analysis shown above in Figure 1B, and further highlighted the importance of separating viral genomes from cellular DNA prior to attempts to identify specific viral binding factors. Following separation, ChIP assays demonstrated strong and specific CTCF binding to the MVM viral genome (Figure 1C; Rad 21, another cellular chromosome binding factor [38] was used as a negative control). Consistent with the ChIP results, we found that, while CTCF displayed a punctate pattern and was found throughout the nucleus of cells at both time points shown, NS1 co-localized with CTCF in both early- and late-stage APAR bodies (Figure 1D, middle and bottom panel respectively). MVM has two potential CTCF binding sites in its genome, one within the NS1 gene and one in the capsid gene (Figure 1E). Inspection of the autonomous parvoviruses H1 and Minute Virus of Canine (MVC), revealed potential CTCF binding sites in the same relative position as MVM, and the dependovirus AAV has a CTCF binding site within its Rep gene (Figure 1E).

**Figure 2:**
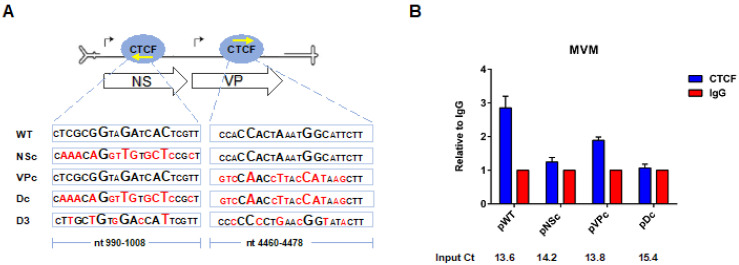
CTCF binding to the single DNA-binding site mutants is reduced, while it is almost completely abolished on the double mutant (**A**) schematic representation of MVM genome showing the positions of transcriptional promoters (solid black arrows), the major open reading frames (arrowed boxes), the relative positions of CTCF binding sites (blue oval shapes) and the nucleotide sequences of the WT and the mutant CTCF binding sites in the NS and VP region. The nucleotides that were mutated within the CTCF binding motifs are shown in red. The yellow arrows show the orientation of the CTCF motifs on the viral genome. (**B**) Human NB324K cells were transfected with the WT or the indicated CTCF-binding site mutants, and harvested at 20 hpt as described in Materials and Methods for whole-cell lysate ChIP using antibodies directed against the cellular factor CTCF or the mouse IgG protein. Samples were analyzed by qPCR as described in Materials and Methods and presented relative to IgG isotype control. Data are presented as mean ± SEM of two individual experiments. Ct values (average of the two experiments) for each plasmid transfection are shown and indicate similar levels of target DNA in the samples.

The sequences of the consensus CTCF binding sites in the MVM genome (RefSeq: NC_001510.1) are shown (Figure 2A). Interestingly, the consensus signals lie on opposite strands of the double stranded transcription template: the consensus NS motif lies in 5′-3′ polarity on the virus minus strand, while the VP motif lies 5′-3′ on the plus strand. To confirm that CTCF bound to the genome at these sites on the double strand replicative form, a series of mutations were made, and these were used as targets for CTCF ChIP experiments. As we could not reproducibly shear the replicating MVM genome during the ChIP procedure, this step was omitted, and so ChIP pull-downs revealed binding to the complete MVM genome. Originally, we attempted to mutate the CTCF binding sites by third nucleotide substitutions, which left the amino acid sequences unchanged; however, these mutations only partially prevented CTCF binding, and so more complete mutations were introduced. These severe mutations of both sites together led to significant loss of CTCF binding over background (Dc; Figure 2A,B). Mutation of the NS site alone reduced CTCF to nearly Dc levels (NSc; Figure 2A,B), while mutation of the VP site retained intermediate binding (VPc; Figure 2A,B). Binding in the single mutants was likely due to binding at the remaining unaltered site, which suggested that CTCF could bind independently to either site, and that binding to the NS site appeared stronger. Unfortunately, mutations needed to prevent CTCF binding destroyed the NS1 open reading frame precluding assessment of their replication. Prior to the further analyses described below, all mutants were marker rescued with wild-type MVM sequences as described in the Materials and Methods to ensure no additional mutations were present.

### 3.2. CTCF-Binding Site Mutants Exhibited a Decrease in Levels of Spliced to Unspliced R1, as Well as Reduced Levels of R2 Relative to R1

**Figure 3:**
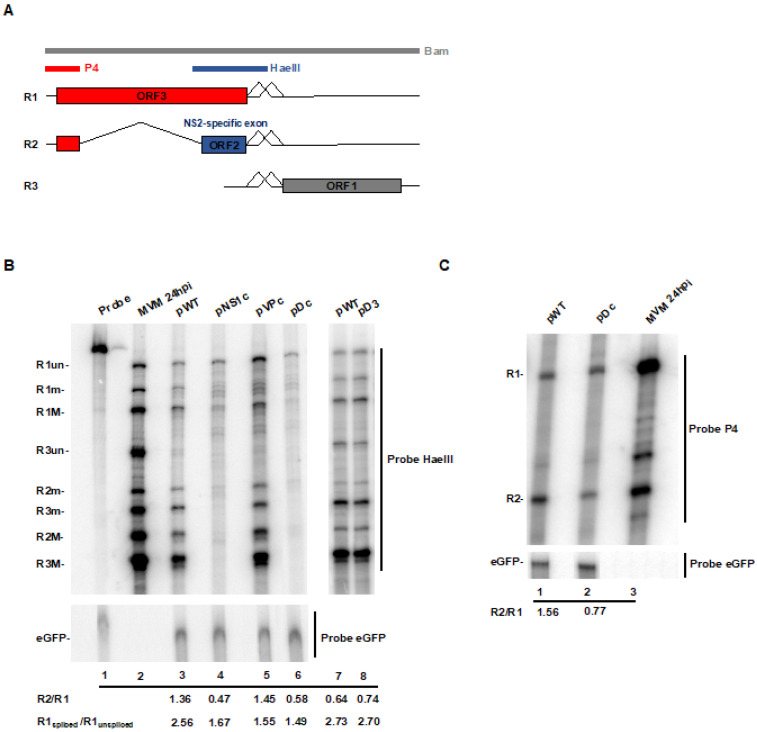
CTCF-binding site mutants exhibit a decrease in levels of spliced to un-spliced R1, and levels of R2 relative to R1. (**A**) Genetic map of MVM showing the three major transcript classes (R1, R2, and R3), the open reading frames that encode the two non-structural (ORF2 and ORF3) and the capsid (ORF1) proteins, and the relative positions of the small and large introns. Approximate locations of the RNase protection viral probes (Bam, P4 and HaeIII) are also indicated. (**B**) Human NB324K cells were infected with MVMp at an MOI of 10 or co-transfected with the indicated plasmids and eGFP. Infected cells were harvested at 24 hpi while transfected cells were harvested at 48 hpt and total RNA was isolated using TRIzol reagent. Samples were processed for RNase protection assay (RPA) using a HaeIII (that detects all viral transcripts) or an eGFP probe, as described in Materials and Methods. The protected bands representing all nine viral mRNA species are indicated to the left. The ratios of total R2 to total R1, and spliced to unspliced R1 are indicated at the bottom of the panel. Ratios represent an average of two independent experiments. (**C**) RPA of total RNA extracted from NB324K cells 24 h post infection or 48 h post transfection with the indicated plasmids using the P4 viral probe, detecting specifically the P4-generated transcripts, or the eGFP probe. The identities of the protected bands are shown on the left. The ratio of R2 to R1 is indicated at the bottom of the panel. Ratios represent an average of two independent experiments.

Following transfection of human NB324K cells, both the Dc double mutant, and the NSc single mutant, were found to generate significantly reduced levels of spliced R1 relative to unspliced R1 RNAs, and reduced levels of R2 relative to R1, as assayed by RNase protection assays [Figure 3B, lanes 4 and 6, respectively (ratios represent an average of two independent experiments)], using the HaeIII probe, which spans the small intron (Figure 3A). As the mutations in the NS region changed the amino acid sequence of NS1, R3 was not generated by either of these mutants. Control transfections of an eGFP expressing plasmid confirmed similar levels of transfection efficiency in these experiments (Figure 3B, bottom panel). Mutants reducing binding within the VP region site alone (VPc), in which CTCF binding to the NS region remained, showed a decrease in splicing to R1, but essentially wild-type patterns of R2 expression (Figure 3B compare lanes 5 with lane 3 and 2). A similar phenotype for the three mutants was also observed following transfection of murine A9 cells. Together, these results indicated that the phenotype of the Dc mutant was primarily due to the mutation in the NS region, and even though CTCF bound at both the NS and VP sites (Figure 2), the individual mutations exhibited different effects. While both mutants exhibited decreased splicing of R1 from the P4-generated pre-mRNA, only the NSc mutation affected subsequent appearance or R2. It is important to note that the NSc mutations fell outside of the affected R2 RNA itself, and did not lie close to any known RNA regulatory element. Additionally, mutation of multiple nucleotides within the NS and VP motifs that did not efficiently disrupt CTCF binding (D3, diagrammed in Figure 2A) had no deleterious effect on RNA processing (Figure 3B, lanes 7, 8). RNase protection assays with a P4 probe, which specifically detects the P4 promoter-generated R1 and R2 transcripts individually, also showed an increased ratio of R2 relative to R1, while the total P4 products were similar for the two. Splicing of R1 pre-mRNA depends upon engagement of the spliceosome at the small intron [30], which was also necessary for exon definition of the upstream NS2-specific exon required for splicing of the large intron and generation of R2 [39]. Thus, our results suggested that CTCF engagement of its MVM binding sites may play a role in processes functioning at the small intron.

### 3.3. CTCF-Binding Site Mutants Resulted in Skipping of the NS2-Specific Exon and Joining of the Large Intron Donor to the Small Intron Acceptors

**Figure 4:**
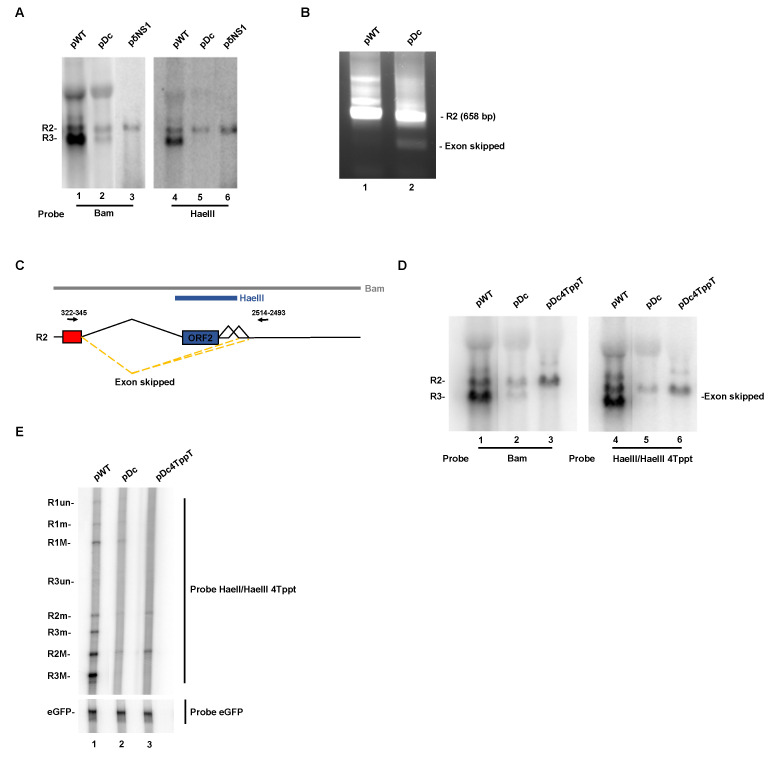
CTCF-binding site mutants resulted in skipping of the NS2-specific exon and joining of the large intron donor to the small intron acceptors, which can be overcome by mutations that improve the polypyrimidine tract of the upstream large intron. (**A**) Human NB324K cells, transfected with the indicated plasmids, were harvested at 48 hpt and total RNA was isolated using TRIzol reagent. Samples were processed for Northern blot analysis using the probes indicated at the bottom of the panel. The identity of the viral RNA species is shown on the left. (**B**) Total RNA extracted from NB324K cells, transfected with the WT or the double CTCF-binding site mutant, was subjected to DNase I treatment for 1 h at 37 °C. First-strand cDNA synthesis was performed on the DNase I-treated RNA samples and the cDNA product was used as a template for downstream PCR analysis as described in Materials and Methods. The identities of the amplified bands were determined by sequencing analysis and are shown to the right. (**C**) Schematic representation of R2 (top) and the exon-kipped product (bottom), generated from the double CTCF-binding site mutant construct. The exon-skipped product skips the NS2-specific exon (ORF2) and joins the large intron donor at nt 514 to the small intron acceptor A1 (nt 2377) or A2 (nt 2399). The PCR primers used to detect the exon-skipped product are indicated at the top of the panel (black arrows). The numbers on top of the arrows represent the location on the viral genome where the primers anneal. Approximate locations of the RNase protection viral probes (Bam and HaeIII) are also indicated. Total RNA extracted from NB324K cells, transfected with the indicated plasmids as well as eGFP, was subjected to Northern blot analysis (**D**) or RNase protection assay (**E**) as described in Materials and Methods using the probes shown at the bottom (**D**) or to the right (**E**) of the panel. The identity of the RNA species are also depicted.

Northern blot analysis of RNA generated in NB324K cells by the double CTCF-binding site mutant Dc revealed a transcript, approximately the size of R3, that hybridized with a whole-genome probe (Figure 4A, lane 2). This was surprising since this mutant, which does not produce wild-type NS1, did not generate the R3 mRNA, as was demonstrated in Figure 3B. A mutant containing a translation termination signal immediately downstream of the NS1 AUG is shown for comparison (Figure 4A, lane 3). Interestingly, the R3-size RNA generated by Dc was not detected in Northern blots using the HaeIII probe, which covers the NS2-specific exon (Figure 4A, lane 5). These results confirmed that this band was not R3, and suggested that the approximate 3 kb size RNA generated by Dc might have been an RNA product spliced at the large intron donor (nt 514) that was joined to a small intron acceptor (Although the large amount of transfected plasmid DNA in these samples makes R1 poorly visible on these gels, it was clearly apparent on the RNase protection gels of these RNAs shown in Figure 3).

To reveal whether such an RNA was in fact made by Dc, we performed non-quantitative RT-PCR analysis of Dc-generated RNA using primers shown in Figure 4C. As shown in Figure 4B, the Dc mutant did generate such a novel spliced product, which is diagrammed in Figure 4C. These cDNAs were cloned and sequence analysis revealed that these spliced products joined the large intron donor at nt 514 to either the small intron acceptor, A1, at nt 2377, or the small intron acceptor, A2, at nt 2399. Inspection of the more quantitative Northern results in Figure 4A suggests that this NS2-specific exon-skipped product was present at approximately half the concentration of R2.

### 3.4. Improvement of the Large Intron Splice Acceptor in the Dc Mutant Led to Increased NS2-Specific Exon Definition and Increased Levels of R2 RNA

If lack of CTCF binding to the MVM genome led to weakening of the large intron acceptor due to loss of definition, for splicing purposes, of the NS2 specific exon, we would expect that improving the large intron acceptor would overcome this deficiency. As can be seen in a Northern blot analysis using the whole genomic probe, strengthening the large intron acceptor polypyrimidine tract with the addition of 4 additional thymidine residues, previously shown to overcome mutations that reduced NS2-specific exon definition [39], led to both a decrease in the exon skipped product and an increase in authentic R2 generated by Dc (Figure 4D, compare lanes 2 and 3). Northern analysis of this RNA using the HaeIII probe confirmed the authenticity of the exon skipped product lost in the left panel of Figure 4 (Figure 4D, compare lane 2 to 5), and revealed enhanced levels of R2. An increase in R2 RNA generated by pDc4Tppt was confirmed by quantitative RNase protection analysis in which expression of an eGFP gene was included as a transfection control (Figure 4E, compare lane 2 to 3).

## 4. Discussion

In surveying the MVM genome for the binding sites of known cellular factors, we noticed consensus CTCF binding sites in the NS and the VP regions of MVM that were conserved in a number of other parvoviruses. Because we previously showed that the replicating MVM genome associates with particular sites of DNA damage on the cellular genome [13], determining whether CTCF specifically bound to MVM required that we separate the viral genome from the cellular genome prior to the cross-linking step during chromatin immunoprecipitation assays. Upon doing so, we could demonstrate specific binding of CTCF to these sites on MVM.

Full disruption of CTCF binding to MVM required destruction of both sites together; destruction of the NS site individually (which retained the VP binding site) reduced binding similarly to the double mutant, while destruction of the VP site (leaving the NS site) retained an intermediate binding phenotype. Because the mutations required to disrupt CTCF binding could not be made without disrupting the NS1 ORF, the mutants could not be assessed directly for replication. However, both the double CTCF binding site mutant Dc, and the NS-alone mutation NSc, showed a dramatic defect in gene expression. These mutants were both deficient in the splicing of the R1 RNA, and they generated relatively less R2 at the expense of a new product, an RNA that joined the large intron donor at nt 514 to one or the other of the small intron acceptors. As previously mentioned, the NSc mutations falls outside of the affected R2 RNA itself, and does not lie close to any known RNA regulatory element. Additionally, mutation of multiple nucleotides within the NS motif that did not efficiently disrupt CTCF binding had no deleterious effect on RNA processing (Figure 3), further implying that this region did not contain a previously unrecognized *cis*-acting RNA processing element. It is interesting that the VPc single mutant, although apparently not deficient in NS2-specific exon definition, still generated less relative spliced R1 RNA. This perhaps suggests that the role of CTCF binding at the individual sites, and their potential interaction, is complex, and may be related to their different orientations on the viral chromosome. Our preliminary results have shown that the exon-skipped RNA product does transit to the cytoplasm, but we could detect no protein product that it generates.

**Figure 5:**
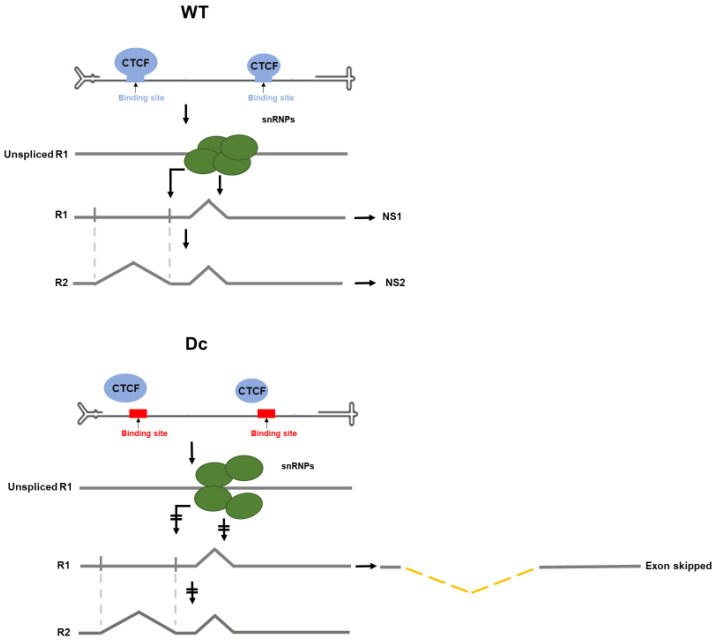
Model depicting a potential mechanism by which CTCF regulates splicing of MVM P4-generated transcripts. Top: A general model of processing of P4-generated pre-mRNAs; CTCF binding to the viral genome likely plays a role in proper engagement of the spliceosome at the small intron, allowing its splicing as well as facilitating its interaction with the upstream large intron acceptor, to define the NS2-specific exon allowing splicing of the large intron. Bottom: In the absence of CTCF binding, R1 would be poorly spliced, and the NS2-specific exon poorly defined, leading to the generation of a novel exon-skipped product.

Interestingly, we observed the newly spliced exon-skipped RNA product before [39,40]. In previous studies that characterized splicing of the P4-generated pre-mRNA, we found that when the NS2-specific exon was poorly defined—either by virtue of its weak large intron acceptor [30], by certain mutations within the NS2-specific exon itself [40], or importantly, by mutation of the downstream small intron [30], an RNA was generated in which the NS2-specific exon was skipped. Because definition of the NS2-specific exon functions to strengthen the adjacent upstream large intron acceptor at nt 1989 [39], improvement, in those mutants, of the large intron acceptor by the addition of four thymidine residues in its polypyrimidine tract overcame the defect in NS2-specific exon definition [39]. These observations, as well as the absence in infected cells, of P4-generated RNAs lacking only the large intron but not the small intron [31], led us to propose a model (diagrammed in Figure 5) in which the spliceosome first engages the R1 pre-mRNA at the small intron, allowing its splicing as well as facilitating its interaction with the upstream large intron acceptor to define the NS2-specific exon allowing splicing of the large intron [39].

In the light of these previous results, the results presented here—that the Dc and NSc mutants exhibited reduced splicing of R1 and generated an exon-skipped product at the expense of R2, which could be suppressed by improvement of the large intron polypyrimidine track—suggested a model in which CTCF binding likely plays a role in proper engagement of the spliceosome at the small intron. In its absence, R1 would be poorly spliced, and the NS2-specific exon poorly defined, leading to the generation of the new exon-skipped product we observe. How interruption of interaction of the spliceosome at the small intron may affect our general model of P4-generated pre-mRNA processing is shown in Figure 5. Binding of CTCF to the site in the NS1 gene appears to play a more significant role in this effect than binding to the site in the capsid gene.

How CTCF binding to MVM functions to play its role in MVM RNA processing is not yet known. CTCF has been shown to have a role in chromosomal architecture, specifically looping of DNA, as well as transcriptional activation, and has been shown to have RNA binding activity [19,20,21,22]. RNA immunoprecipitation experiments, done as we have previously described [41] did not demonstrate CTCF binding to MVM RNA. It was first reported that DNA-bound CTCF regulates alternative pre-mRNA splicing by mediating RNA Polymerase II pausing, allowing the inclusion of upstream weak exons [23]. A more recent report, however, suggested that CTCF regulation of alternative splicing of human papillomavirus early genes was more complicated. Specifically, it was found, similar to the results reported here, that loss of CTCF binding to the viral genome resulted in both increased levels of unspliced transcripts and an alteration of splice site usage upstream of the CTCF binding site, with a significant reduction of a specific alternatively spliced product [29]. Thus, it is possible that CTCF binding affects RNA processing through modulation of the elongating transcription complex. ChIP assays of RNA pol II on the MVM P4 promoter showed no reproducible difference between the Dc mutant and wildtype MVM. However, it is well known that RNA processing factors bind to the extending RNA polymerase at its CTD [42], and our assays would not have distinguished if the composition of the complexes engaging the Dc P4 promoter and wild-type P4 differed. Perhaps relevantly, we have previously shown that when the AAV2 P40 promoter and the AAV5 P5 promoter were replaced with either the HIV LTR or the CMV promoter, the RNA generated by these constructs was processed differently [43,44]. 

Importantly, it has been shown that the ratio of R2:R1 is exquisitely critical for successful MVM infection. Even small changes in this ratio can have large effects on replication [45]. Thus, the role of CTCF in controlling the ratio of R2 to R1 would be predicted to have significant effects on replication.
